# A Safe Method for Early Rehabilitation of Articular Fracture at the Base of Thumb Metacarpal Bone

**DOI:** 10.1155/2021/6632211

**Published:** 2021-02-08

**Authors:** Yunus Oc, Bekir Eray Kilinc, Ali Varol, Adnan Kara

**Affiliations:** ^1^Medilife Health Group, Bagcilar Hospital, Department of Orthopaedics and Traumatology, Istanbul, Turkey; ^2^Health Science University Istanbul Fatih Sultan Mehmet Training and Research Hospital, Department of Orthopaedics and Traumatology, Istanbul, Turkey; ^3^Health Ministry, Silopi State Hospital, Department of Orthopaedics and Traumatology, Sirnak, Turkey; ^4^Medipol University Istanbul, Department of Orthopaedics and Traumatology, Istanbul, Turkey

## Abstract

**Background:**

To evaluate the clinical and radiological results of closed reduction, distraction using an external fixator, and percutaneous fixation in patients with Bennet and Rolando fractures.

**Methods:**

Patients over 18 years of age, who had isolated fracture at the base of the first metacarpal bone, had no previous functional limitations and pain complaints, were regularly followed up, and had fixation using K-wire combined with an external fixator, were included. Arthrosis was evaluated according to Eaton and Littler classification. Pain intensity was evaluated using the visual analogue scale (VAS) on a 0–10 scale. Furthermore, patients were questioned regarding limitations in their daily activities and hobbies. Pinch and grasp strengths were evaluated.

**Results:**

Thirteen of the patients were male and five were female, with a mean age of 31.5 ± 12.5 years. The surgical procedure was performed on the right extremity in 12 patients and left extremity in six patients. Twelve patients were found to have Bennet fractures, whereas six patients had Rolando fractures. The mean follow-up period of the patients was found to be 29.6 ± 5.4 months. The VAS score was rated as 2 in one patient and 1 in one patient. Other patients had a pain VAS score of 0. The mean Quick-DASH score was calculated to be 1.20. No statistical difference was found in pinch strength between the two extremities (*p* > 0.05). No difference was observed in terms of the range of motion (*p* > 0.05).

**Conclusion:**

Fixation using K-wire combined with an external fixator has more benefits than its disadvantages and is superior to other methods in the intra-articular fractures of the first metacarpal bone.

## 1. Introduction

The thumb is an essential part of hand function in dexterity, particularly for pinching and grasping. The trapeziometacarpal (TMC) joint serves as a pivot point in the thumb column and allows simple and complex movements to be performed.

Proximal metacarpal fractures usually occur due to exposure to a moderate trauma, and their incidence is higher in the working adult population. These injuries can present as both extra- and intra-articular injuries, both causing problems with stability at the fracture level [1, 2]. Since these fractures are generally displaced at the time of presentation, they are risky in terms of stiffness, instability, and progression to arthrosis in the carpometacarpal joint.

Intra-articular fractures of the first metacarpal proximal end, which are generally referred to as Rolando and Bennet fractures, account for 1.4% of hand fractures [3, 4]. Treatment of these fractures is still controversial. In addition to conservative treatment, many different surgical treatment modalities have been defined. The common goal of all treatment methods is the high-quality restoration of the articular surface [1, 2].

The hypothesis of the present study is that the initiation of movement at the same time in the first carpometacarpal joint restored by distraction and percutaneous pinning method with an external fixator will not create functional restrictions during the treatment process and will reduce posttreatment complications.

This study aimed to evaluate the clinical and radiological results of closed reduction, distraction using an external fixator, and percutaneous fixation in patients with intra- and extra-articular fractures at the base of the first metacarpal bone.

## 2. Materials and Methods

This retrospective study conducted over a two-year period included all Bennett-type or Rolando-type intra-articular fractures at the base of the first metacarpal bone. Analysis of patient records revealed that mainly young males suffered these fractures. The study was conducted at a single institution between 2018 and 2020. This study was conducted with the approval of the Institutional Review Board and was in line with the ethical principles of the Declaration of Helsinki. The reference number for the ethics committee approval was 2020/344-67.

Patients over 18 years of age, who had isolated fracture at the base of the first metacarpal bone, had no previous functional limitations and pain complaints, and were regularly followed up, were included.

Patients were evaluated with standard anteroposterior (AP) and lateral radiographs of the hand. The thumb column was placed parallel to the antepulsion-retropulsion axis in the AP view and the flexion-extension axis in the lateral view.

### 2.1. Surgical Technique

**Figure 1:**
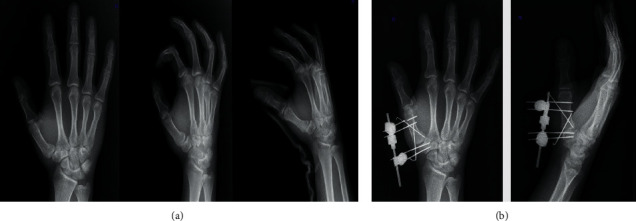
(a) Preop AP/lateral and oblique X-ray views of Bennet fracture. (b) Postop AP/lateral X-ray views of Bennet fracture.

General anaesthesia was induced in all patients. Closed reduction was achieved under fluoroscopy with longitudinal traction of the thumb combined with abduction-extension maneuver and metacarpal pronation. Two Schanz pins were applied in the trapezium and two Schanz pins to the distal of the first metacarpal bone. Then, alignment was achieved by placing an external fixator. Using the ligamentotaxis effect of the fixator (Unilateral finger fixator, Design Med, Turkey), a 2 mm distraction was applied to the joint. The joint restoration was ensured with Kirschner wires (K-wire) of 1.4 mm in the appropriate configuration according to the intra-articular fracture fragment of each patient. Intra-articular step-off was accepted as 1 mm. Thumb movements were examined under fluoroscopy to check the stability of the fixation (Figure 1).

Rehabilitation was started on the first postoperative day. The patients were evaluated in the 1st, 3rd, 6^th^, and 24th months. The K-wires and fixator were removed under local anaesthesia since the consolidation was seen in the control graph taken in the first month. Wound healing and functional healing were evaluated at the third- and fifth-month controls. Patients were evaluated in terms of pain and function at the 24th month. Pain intensity was evaluated using the visual analogue scale (VAS) on a 0–10 scale. Furthermore, patients were questioned regarding limitations in their daily activities and hobbies. Pinch and grasp strengths were evaluated using a Hand Dynamometer and Pinch Gauge (Fabrication Enterprises Inc., New York, NY, USA).

The values of the operated and healthy sides of the patients were compared to measure the pinch and grasp strengths. A difference of 20% with the dominant hand was considered significant [5].

In radiographs, posttraumatic arthrosis was evaluated according to van Niekerk and Owens modification of the Eaton and Littler classification: Stage I: no clear arthritic changes, Stage II: osteophytes smaller than 2 mm, Stage III: osteophytes larger than 2 mm or joint narrowing, and Stage IV: joint space more or less disappeared [6, 7]. Statistical analyses were performed using Statistical Package for Social Sciences (SPSS) version 14.0. A nonparametric Mann–Whitney test was used to compare the functional results between the injured and noninjured hand.

General anaesthesia was induced in all patients. All patients were hospitalized for one day following the operation, and the external fixator was kept in place for six weeks in all patients. At the sixth week, fixators and K-wires were extracted under local anaesthesia under polyclinic conditions. Outpatient clinic controls were made in all patients at the third, sixth, 12th, and 24th weeks. At the 24-week controls, VAS was applied to all patients to measure the pain intensity, and the shortened version of the disabilities of the arm, shoulder, and hand questionnaire (Quick-DASH) was performed to evaluate physical functions subjectively.

## 3. Results

A total of 18 patients meeting the study criteria, who had proximal end intra-articular extension fracture at the first metacarpal bone and were treated with an external fixator and percutaneous pinning between 2016 and 2019, were included in the study. Thirteen of the patients were male and five were female, with a mean age of 31.5 ± 12.5 years. The surgical procedure was performed on the right extremity in 12 patients and left extremity in six patients. The affected extremity was the dominant extremity in 14 patients. The mechanism of injury was occupational accidents in five patients, traffic accident in four patients, battery in two patients, and simply falling in seven patients. Twelve patients were found to have Bennet fractures (according to the Gedda classification: five patients had type 1, six patients had type 2, and one patient had type 3), whereas six patients had Rolando fractures. The mean follow-up period of the patients was found to be 29.6 ± 5.4 months (Table 1).

The pain VAS score was rated as 2 in one patient and 1 in one patient. Other patients had a pain VAS score of 0. The mean Quick-DASH score of the patients was calculated to be 1.20. The pinch and grasp strengths of the patients were measured by evaluating the opposite (nonaffected) extremity, and the mean pinch strength was found to be 94% whereas the mean grasp strength was 98%. There was no statistical difference between the two extremities (*p* > 0.05). No difference was observed between the operated and nonoperated sides in terms of the range of motion (ROM) (*p* > 0.05). Similarly, there was no difference between the affected and nonaffected sides in terms of ROM (*p* > 0.05). Full opening was observed in the interphalangeal (IP), metacarpophalangeal (MCP), and carpometacarpal (CMC) joints.

**Figure 2:**
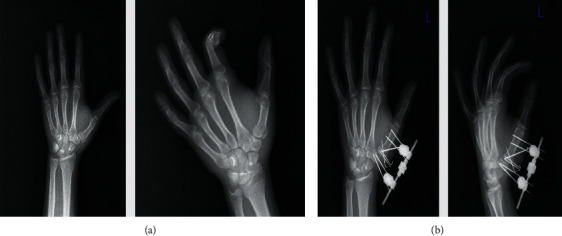
(a) Preop AP and oblique X-ray views of Rolando fracture. (b) Postop AP/lateral X-ray views of Rolando fracture.

Joint arthrosis was evaluated in the radiographs taken at the 24th week. According to Eaton and Littler's classification [8], 16 patients had stage I arthrosis and two had stage II arthrosis. Two patients were observed to have pin tract infection, which regressed with one-week antibiotic treatment (Figure 2).

## 4. Discussion

Distraction using an external fixator and K-wire fixation provide safe stability for early postoperative rehabilitation of the fractures at the base of the first metacarpal bone. These methods are useful in early rehabilitation of the joint movements and full recovery of functional capacity. The high bone union rates and low rate of joint degeneration obtained by the method applied in the present study indicate that this method is effective.

Since the time when Rolando and Bennet fractures were first identified, various treatment modalities from conservative treatment to open reduction and fixation and even arthroscopic-assisted fixation have been presented for these fractures. Reduction of proximal fractures of the 1stmetacarpal bone with intra-articular extension and maintaining the reduction are difficult due to the complexity of the forces acting on the joint [9]. Therefore, various treatment modalities are used in the treatment of fractures at the base of the first metacarpal bone. The most common ones among these treatment modalities are closed reduction and plaster cast application; closed reduction and pinning; closed reduction and external fixator application; open reduction and fixation with plate or screw; and closed reduction, external fixator application, and pinning [9–12]. Closed reduction and plaster cast application is not preferred since it does not allow early mobilization and fails to prevent arthrosis in the joint. Since closed reduction and external fixator application alone is not sufficient for joint surface restoration, the possibility of arthrosis development in the joint is higher in the long term. Kontakis et al. [13] performed closed reduction and external fixator application in 11 patients and reported that there was joint arthrosis in four of these patients and one of them was severe. Open reduction and plate fixation is a preferred treatment modality since it provides early mobilization and stable fixation. Development of soft tissue problems, devascularization of fracture fragments, and collateral ligament and tendon injuries are more common in this method. In a study by Mumtaz et al. [14], the implant was required to be removed in four of nine patients, who underwent open reduction and plate osteosynthesis, due to local tenderness and pain, and soft tissue problem and superficial infection were reported in two patients. Closed reduction and external fixator and pinning is presented as a much safer method for fractures of this region in terms of both joint restoration and early mobilization. In a case series by Houshain and Jing [12] involving 16 patients, two pin tract infections were reported as a complication, and the authors reported that they achieved excellent results in 12 of the 16 patients and good results in four. In the present study, change was observed in VAS scores and joint arthritis in only two of the patients. The fixation preferred in the current study is reliable enough to allow early mobilization without causing loss of reduction. Thus, complications such as joint stiffness and future arthrosis can be minimized. No limitation was observed in joint movements of the patients included in this study. Achieving better and safer results with the reduction of the fractures of this area through the effect of ligamentotaxis and restoration using K-wires in the joint, compared to others, will make it to be preferred more often.

The small number of patients and its retrospective design are among the limitations of the present study. The sample size of the present study determined using power analysis is strong, which is compatible with the literature. Another limitation is the absence of a control group. We believe that it will be difficult to create a control group since there are various options in the treatment of the first metacarpal basis fractures. Therefore, there is a need for a large prospective series with long-term follow-up to determine optimal treatment guidelines.

Compared to the traditional treatment methods, such as fixation with a splint or K-wire fixation, fixation using K-wire combined with an external fixator is superior in maintaining anatomic reduction of displaced and severely communated fractures [12, 13]. The surgical method used in the present study eliminates the need for large soft tissue in cases where open reduction and internal fixation is indicated and prevents dissection for the fixation of the fractured part and complications such as avascular necrosis. We can recommend this method as a primary and definitive treatment in cases where conservative treatments and other surgical interventions fail.

The following are among the main advantages of this method: providing good functional results, causing fewer complications, allowing early mobilization, and not requiring neither reoperation nor physical therapy. Living with an external fixator though for a short time and pin tract infections are the disadvantages of this method.

## 5. Conclusions

We believe that this method, which has more benefits than its disadvantages, is superior to other methods in the intra-articular fractures of the first metacarpal bone.

## Figures and Tables

**Table 1:** Demographic data of the patients.

Patient	Age	Sex	Side	Type of fracture	Injury type	f/u	Complication	VAS	Quick-dash	Pinch %	Grasp	Eaton–Littler classification

1	28	M	R	Bennet	İA	26	—	0	0	96	96	Stage 1
2	26	M	R	Bennet	TA	27	—	0	0	96	100	Stage 1
3	38	M	L	Rolando	Fall	30	—	1	6, 8	90	96	Stage 2
4	44	F	R	Bennet	DT	26	PTE	0	0	98	100	Stage 1
5	36	M	R	Rolando	İA	24	—	0	0	94	98	Stage 1
6	29	F	R	Bennet	Fall	28	—	0	0	94	100	Stage 1
7	34	M	L	Bennet	TA	32	—	0	0	94	96	Stage 1
8	40	M	R	Bennet	İA	31	—	0	0	96	98	Stage 1
9	26	F	R	Rolando	DT	34	—	0	0	94	100	Stage 1
10	42	M	R	Bennet	Fall	28	—	0	0	94	98	Stage 1
11	30	M	L	Bennet	Fall	32	—	0	0	96	100	Stage 1
12	26	M	R	Rolando	İA	24	PTE	0	0	94	98	Stage 1
13	19	F	L	Bennet	Fall	34	—	0	0	96	100	Stage 1
14	24	M	R	Rolando	TA	27	—	0	0	94	100	Stage 1
15	21	M	R	Bennet	TA	35	—	0	0	94	96	Stage 1
16	39	F	L	Bennet	Fall	36	—	0	0	96	98	Stage 1
17	41	M	R	Rolando	İA	28	—	2	11, 4	88	90	Stage 2
18	25	M	L	Bennet	Fall	32	—	0	0	96	100	Stage 1

İA: industrial accident, TA : traffic accident, DT : direct trauma, PTE : pin Tract infection.

## Data Availability

Data are available upon request.
